# Myocardial ischaemic burden assessed by three-dimensional perfusion CMR - comparison with Myocardial Perfusion Scintigraphy

**DOI:** 10.1186/1532-429X-15-S1-P175

**Published:** 2013-01-30

**Authors:** Roy Jogiya, Geraint Morton, Mark Peterzan, Kalpa De Silva, Sebastian Kozerke, Eike Nagel, Stephen R Underwood, Sven Plein

**Affiliations:** 1 Kings College London, London, UK; 2 Imperial College London & Royal Brompton & Harefield NHS Foundation Trust, London, UK; 3 Leeds University, Leeds, UK; 4 ETH Univesity, Zurich, Switzerland

## Background

Myocardial ischaemic burden assessed by myocardial perfusion scintigraphy (MPS) is commonly used to risk-stratify patients with suspected coronary artery disease (CAD). Accurate estimation of ischaemic burden by CMR with two-dimensional imaging is limited by incomplete myocardial coverage to a small number (usually three) of non-contiguous sections. The aim of this study was to compare ischaemic burden on 3D myocardial perfusion CMR with technetium-99m-tetrofosmin myocardial perfusion scintigraphy (MPS).

## Methods

Forty-six consecutive patients with known or suspected CAD who were referred clinically for MPS underwent 3D CMR perfusion at rest and during adenosine stress as well as late gadolinium enhancement (LGE) on a 3T Philips Achieva TX system. The CMR perfusion images were scored visually by an experienced observer who was blinded to the MPS findings. A 17 segment model was used with a defect scale of 0-4 based on trans-murality of the stress perfusion defect: 0 none; 1 mild (25-49%); 2 moderate (50-74%); 3 severe (75-100%); and 4 absent (thinning with transmural LGE). Resting scores were assigned from the trans-murality of scar on LGE images. The summed stress, rest and difference scores were expressed as a percentage of the theoretical maximum score of 68. MPS images were analysed in a similar manner using segmental defect scores from 0-4 (normal - absent tracer uptake).

## Results

**Figure 1:**
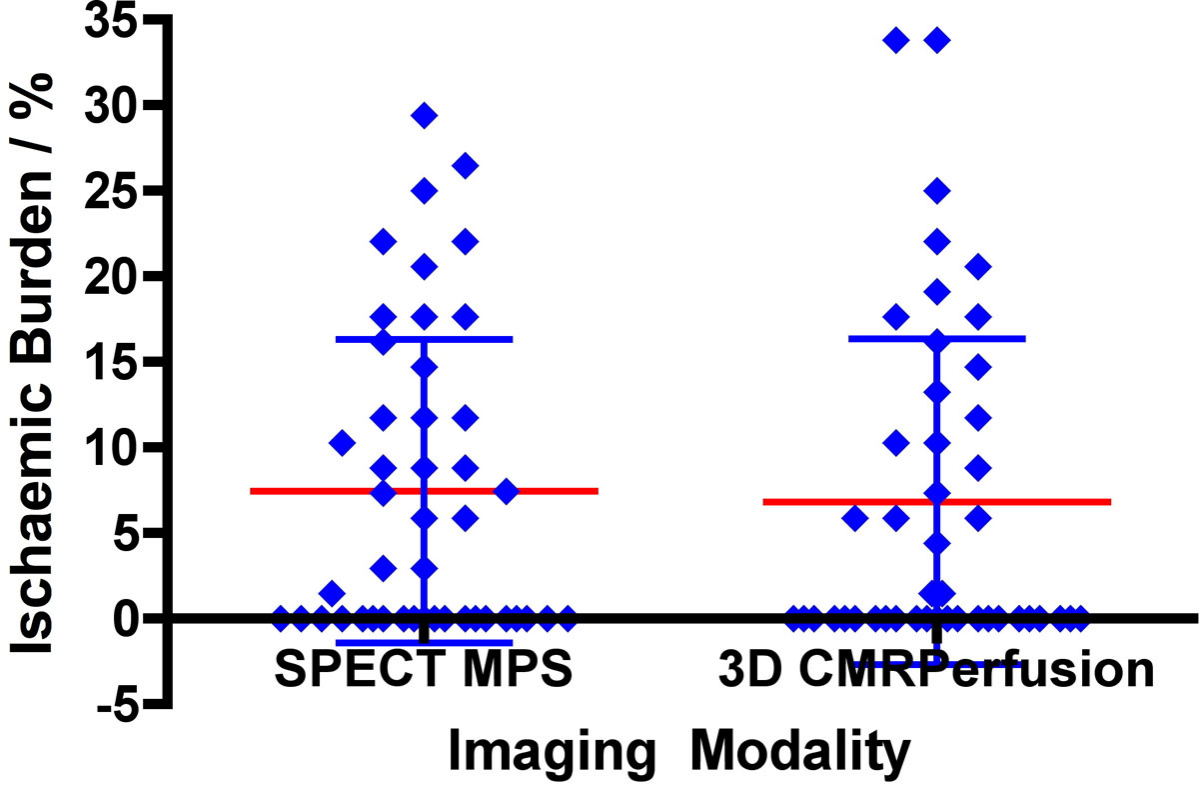
Ischaemic burden calculated from the two different imaging modalities

One patient was excluded from analysis because of significant motion artefact on CMR. 3D myocardial perfusion CMR and MPS agreed in 38 of the remaining 45 patients for the detection of any inducible ischaemia. The mean percentage of inducible ischaemia for MPS was 7.5% SD 8.86% and for 3D CMR perfusion 6.8% SD 9.51% (P=0.59) (Figure 1). When analyzing only the patients in whom the two tests agreed, there was a strong correlation in ischaemic burden between the techniques (R2=0.81, P<0.0001).

## Conclusions

3D myocardial perfusion CMR agrees well with MPS for the detection of coronary artery disease. Both techniques detect similar ischaemic burden. 3D myocardial perfusion CMR offers a promising alternative method of detecting the presence and severity of ischaemia with the added benefits of higher spatial resolution and no burden of ionising radiation.

## Funding

Prof. Plein is funded by British Heart Foundation fellowship FS/10/62/28409 and receives research grant support from Philips Healthcare.

Prof. Kozerke receives funding from the Swiss National Science Foundation (grant number CR3213_132671/1) and research support form Bayer (Switzerland) AG. Prof. Nagel receives grant support from Bayer Healthcare and Philips Healthcare.

